# The Application of Multi-Locus GWAS for the Detection of Salt-Tolerance Loci in Rice

**DOI:** 10.3389/fpls.2018.01464

**Published:** 2018-10-04

**Authors:** Yanru Cui, Fan Zhang, Yongli Zhou

**Affiliations:** Institute of Crop Sciences/National Key Facility for Crop Gene Resources and Genetic Improvement, Chinese Academy of Agricultural Sciences, Beijing, China

**Keywords:** multi-locus, GWAS, QTNs, salt tolerance, rice

## Abstract

Improving the salt-tolerance of direct-seeding rice at the seed germination stage is a major goal of breeders. Efficiently identifying salt tolerance loci will help researchers develop effective rice breeding strategies. In this study, six multi-locus genome-wide association studies (GWAS) methods (mrMLM, FASTmrMLM, FASTmrEMMA, pLARmEB, pKWmEB, and ISIS EM-BLASSO) were applied to identify quantitative trait nucleotides (QTNs) for the salt tolerance traits of 478 rice accessions with 162,529 SNPs at the seed germination stage. Among the 371 QTNs detected by the six methods, 56 were identified by at least three methods. Among these 56 QTNs, 12, 6, 7, 4, 13, 12, and 12 were found to be associated with SSI-GI, SSI-VI, SSI-MGT, SSI-IR-24h, SSI-IR-48h, SSI-GR-5d, and SSI-GR-10d, respectively. Additionally, 66 candidate genes were identified in the vicinity of the 56 QTNs, and two of these genes (LOC_Os01g45760 and LOC_Os10g04860) are involved in auxin biosynthesis according to the enriched GO terms and KEGG pathways. This information will be useful for identifying the genes responsible for rice salt tolerance. A comparison of the six methods revealed that ISIS EM-BLASSO identified the most co-detected QTNs and performed best, with the smallest residual errors and highest computing speed, followed by FASTmrMLM, pLARmEB, mrMLM, pKWmEB, and FASTmrEMMA. Although multi-locus GWAS methods are superior to single-locus GWAS methods, their utility for identifying QTNs may be enhanced by adding a bin analysis to the models or by developing a hybrid method that merges the results from different methods.

## Introduction

A genome-wide association studies (GWAS) represents a powerful option for the genetic characterization of quantitative traits, and has been widely used for analyzing agronomic traits related to plants. Numerous genetic variants for complex traits have been identified based on single-locus GWAS methods, such as empirical Bayes, efficient mixed model association (EMMA), genome-wide efficient mixed linear model association (GEMMA), settlement of mixed linear model under progressively exclusive relationship (SUPER), and mixed linear model (MLM) (Kang et al., 2008; Zhou and Stephens, 2012; Wang et al., 2014, 2016a). Although the statistical power of quantitative trait nucleotide (QTN) detection improves after controlling the polygenic background, most of the small effects associated with complex traits are still not captured by single-locus GWAS methods.

In a single-locus GWAS model, markers are tested individually in a one-dimensional genome scan. Moreover, the multiple test correction for the critical value of a significance test should be considered. Bonferroni correction is widely used to modify the threshold value to control the false positive rate (FPR). However, this type of correction method is so conservative that true QTNs may be eliminated. Therefore, the best way to solve this problem is to develop a multi-locus GWAS method that does not require a multiple test correction. Multi-locus GWAS methods involve a multi-dimensional genome scan, in which the effects of all markers are simultaneously estimated. Many penalized multi-locus GWAS methods have been developed, including the least absolute shrinkage and selection operator (LASSO), empirical Bayes LASSO, and adaptive mixed LASSO (Yi and Xu, 2008; Cho et al., 2009, 2010; Wu et al., 2009; Ayers and Cordell, 2010; Wang et al., 2010; Giglio and Brown, 2018). These methods can minimize some marker effects to zero when the number of single nucleotide polymorphisms (SNPs) is not much larger than the sample size. However, the rapid development of sequencing technologies has enabled the detection of many SNPs (i.e., the number of SNPs is hundreds of times larger than the sample size). Thus, the available methods for minimizing marker effects are ineffective. One option for addressing this issue involves decreasing the number of SNPs. Dr. Zhang’ lab developed an R package called mrMLM, which includes the following six multi-locus GWAS methods: mrMLM, FASTmrMLM, FASTmrEMMA, pLARmEB, pKWmEB, and ISIS EM-BLASSO. All of these methods involve two-step algorithms. During the first step, a single-locus GWAS method is applied to scan the entire genome, and putative QTNs are detected according to a less stringent critical value, such as *P* < 0.005 or *P* < 1/*m*, where *m* is the number of markers. During the second step, all selected putative QTNs are examined by a multi-locus GWAS model to detect true QTNs (Wang et al., 2016a,b; Tamba et al., 2017; Zhang et al., 2017; Ren et al., 2018; Wen et al., 2018a,b; Zhang and Tamba, 2018). The mrMLM package solves the problem associated with co-factor selection in the multi-locus GWAS model when there are many markers.

Rice (*Oryza sativa* L.), which is one of the most important cereal crops worldwide, is sensitive to salt stress. With the increasing salinization of soils, salt stress is becoming a key abiotic factor limiting rice production that rice breeders must overcome (Hu et al., 2012). Developing salt-tolerant rice cultivars is an efficient way to minimize crop loss. Over the past several years, high density SNPs have been used to detect variants with GWAS methods to improve rice varieties (Han and Huang, 2013; Chen et al., 2014; Yang et al., 2014; Wei et al., 2017). However, most traits related to abiotic stress tolerance are controlled by several polygenes that are undetectable in single-locus GWAS models (Lee et al., 2003; Cui et al., 2015). Therefore, we should apply multi-locus GWAS methods to identify loci related to salt tolerance. In this study, 478 rice accessions, each with seven salt stress susceptibility index (SSI)-related traits, and 162,529 SNPs were used to conduct a multi-locus GWAS. Our objectives were to identify the significant QTNs related to salt tolerance and provide recommendations regarding the selection of a multi-locus GWAS method by comparing the differences among the six multi-locus methods included in the mrMLM package.

## Materials and Methods

### Rice Phenotypic Data Related to Salt Tolerance

We analyzed 478 rice accessions from 46 countries and regions regarding seven salt tolerance-related traits at the seed germination stage in a multi-locus GWAS. Phenotypic data were collected for control and stress-treated plants incubated in a growth chamber, with two independent experiments conducted for the control and stress treatments. Each independent experiment involved a randomized block design with two replicates. The dataset was published by Shi et al. (2017), and the seven salt tolerance-related traits were VI, GI, germination rate (GR) at days 5 and 10, MGT, and imbibition rate (IR) at 24 and 48 h. All salt tolerance-related traits were measured for plants treated with 60 mM NaCl or water (control) as follows: IR (mg/g) was calculated as *IR* = (*W*_2_ - *W*_1_)/*W*_1_ × 1000 at 24 and 48 h after starting the incubation, where *W*_1_ represents the dry seed weight and *W*_2_ represents the imbibed seed weight; GR was calculated as *GR* = *N*_t_/*N*_0_ × 100% at days 5 and 10, where *N*_t_ is the number of germinated seeds at day *t* and *N*_0_ is the total number of seeds; GI was calculated as *GI* = ∑ (*G*_t_/*T*_t_), where *G*_t_ is the accumulated number of germinated seeds at day *t* and *T*_t_ is the time (in days); MGT was calculated as *MGT* = ∑ *T*_i_*N*_i_/∑ *N*_i_, where *N*_i_ is the number of newly germinated seeds at day *t* and *T*_i_ is the time (in days); VI was calculated as *VI* = *GI* × *SL*, where *SL* is the average shoot length of 10 germinated seeds at day 10. The salt tolerance level of rice during the germination stage was estimated with the following equation: *SSI* = (1 - *Y*_s_/*Y*_p_)/*D*, where *Y*_s_ is the performance of an individual under the stress condition, *Y*_p_ is the performance of an individual under the normal condition, and *D* is the stress intensity, which was calculated as *D* = 1 - (∑ *Y*_s_/∑ *Y*_p_). Finally, 21 traits were included in this study. The abbreviated names of these 21 traits are provided in the abbreviations list.

### Genotyping and Multi-Locus GWAS

The 478 rice accessions analyzed in this study were from the 3K rice genome project. The 3K rice genome project 404K coreSNP dataset from the Rice-Seek Database was downloaded from http://snp-seek.irri.org/_download.zul (Alexandrov et al., 2015). We used the PLINK program (version 1.9) (Purcell et al., 2007) to obtain a subset of 162,529 SNPs with a minor allele frequency > 5% and a missing data ratio < 0.1 for association analyses. The kinship matrix (*K* matrix) was calculated based on the genotype marker information described by Xu (2013). The mrMLM package, including six multi-locus GWAS methods, was downloaded from http://cran.r-project.org/web/packages/mrMLM/index.html. Default values were used for all parameters.

### Annotation of Candidate Genes and Pathway Enrichment Analysis

Synonymous and non-synonymous SNPs and SNPs associated with large-effect changes were annotated using the snpEff program (version 4.0) (Cingolani et al., 2012) based on the gene models of the annotated Nipponbare reference genome (IRGSP 1.0) (Kawahara et al., 2013). All putative SNPs located within genes and annotation details have been published (Kawahara et al., 2013). Enriched gene ontology (GO) terms and Kyoto Encyclopedia of Genes and Genomes (KEGG) pathways were identified using the agriGO (version 2.0) (Tian et al., 2017) and EXPath 2.0 (Chien et al., 2015) programs, respectively.

## Results

### Heritability and Variance

**FIGURE 1:**
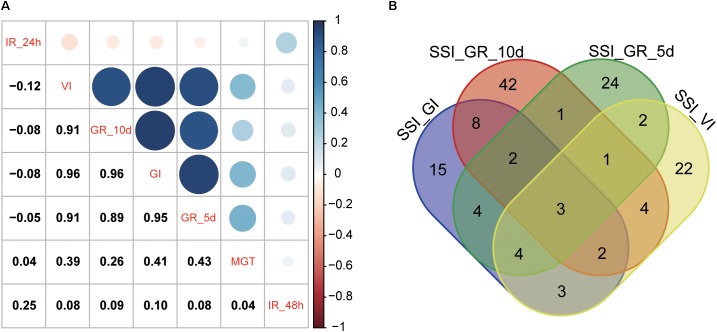
Correlation among SSI-related traits **(A)** and a Venn diagram of the QTNs for four SSI-related traits **(B)** estimated by a multi-locus GWAS.

The heritability and residual errors estimated by the six multi-locus GWAS methods are presented in **Table 1**. The narrow sense heritability ranged from 0.17 for S_MGT and 0.57 for S_IR_48h. A comparison of the residual errors among the six multi-locus GWAS models revealed that the residual error estimated by FASTmrEMMA was the largest under the normal condition when the phenotypic variation was larger than 10. Under the salt stress condition, the largest residual errors for traits S_IR_24h and S_IR_48h were observed from FASTmrEMMA. Regarding the SSI-related traits, the largest residual error was estimated by FASTmrEMMA. The salt tolerance level was evaluated according to the SSI-related traits. Lower SSI values indicated a higher tolerance to salt stress. The results of the correlation analyses of the seven SSI-related traits are presented in **Figure 1A**. There were significant positive correlations among SSI_VI, SSI_GR_5d, SSI _GR_10d, and SSI_GI. The correlation coefficients between SSI-VI and the other three SSI-related traits, namely SSI_GR_5d, SSI_GR_10d, and SSI_GI, were 0.91, 0.91, and 0.96, respectively. Meanwhile, the correlation coefficients for SSI_GR_5d, SSI_GR_10d, and SSI_GI were 0.89, 0.95, and 0.96, respectively. The high correlation among the four SSI-related traits implied that some novel loci might be simultaneously detected for different traits.

**Table 1:** Phenotypic variance, estimated residual error, and heritability of 21 rice traits.

Trait	PV	Heritability (%)	Residual error					
			
			FASTmrEMMA	FASTmrMLM	ISIS EM-BLASSO	mrMLM	pKWmEB	pLARmEB
S_GI	0.17	42	0.13	0.13	0.11	0.10	0.12	0.13
S_VI	2.64	41	2.11	1.63	1.39	1.55	1.48	1.70
S_MGT	1.03	17	0.85	0.74	0.72	0.86	0.66	0.85
S_IR_24h	53351.6322	51	36552.52	23546.99	22673.23	26406.72	21410.21	24658.42
S_IR_48h	52655.13	56	32734.46	24011.68	21900.10	23275.70	24914.75	19703.57
S_GR_5d	1158.11	34	900.85	935.09	690.32	765.26	746.93	815.23
S_GR_10d	1233.41	35	844.53	961.70	893.90	796.73	719.22	907.11
N_GI	0.07	43	0.05	0.04	0.04	0.04	0.04	0.04
N_VI	5.84	34	3.72	3.10	3.00	3.86	2.72	3.18
N_MGT	0.82	50	0.57	0.48	0.36	0.41	0.43	0.49
N_IR_24h	63786.10	55	41714.56	30057.04	26812.22	28640.95	30489.47	31072.55
N_IR_48h	66260.99	48	44506.99	30736.87	23802.86	34238.02	32400.73	26220.37
N_GR_5d	452.67	27	326.57	307.38	233.09	330.44	267.86	285.44
N_GR_10d	86.61	31	66.58	58.11	48.46	59.51	51.38	52.48
SSI_GI	0.41	33	0.31	0.25	0.22	0.27	0.25	0.28
SSI_VI	0.10	22	0.08	0.06	0.05	0.08	0.06	0.07
SSI_MGT	4.07	32	3.61	3.02	2.57	2.75	2.66	3.22
SSI_IR_24h	199.2613	15	176.41	164.03	143.96	23.17	121.67	173.06
SSI_IR_48h	13.76	42	10.59	8.46	6.62	7.95	7.18	8.67
SSI_GR_5d	0.54	27	0.44	0.40	0.32	0.39	0.30	0.38
SSI_GR_10d	0.4509	34	0.34	0.31	0.23	0.30	0.24	0.28


### QTNs Associated With Salt Tolerance at the Germination Stage Identified by a Multi-Locus GWAS

Using the six multi-locus GWAS methods in the mrMLM package (**Supplementary Table S1**), we identified 371 significant QTNs for the salt tolerance-related traits (SSI-VI, SSI-GR, SSI-IR, SSI-MGT, and SSI-GI) based on a logarithm of odds (LOD) threshold of ≥3. Of these QTNs, 41, 41, 27, 63, 56, 41, and 151 were found to be associated with SSI-GI, SSI-VI, SSI-MGT, SSI-IR-24h, SSI-IR-48h, SSI-GR-5d, and SSI-GR-10d, respectively, with the QTNs explaining 0.57 ∼ 9.80, 0.54 ∼ 8.97, 0.64 ∼ 8.21, 0.01 ∼ 4.94, 0.37 ∼ 8.93, 0.9 ∼ 6.72, and 0.7 ∼ 6.08 (%) of the phenotypic variations, respectively [i.e., phenotypic variation explained (PVE) values] (**Supplementary Table S1** and **Supplementary Figure S1**). Additionally, 3, 9, and 22 QTNs were associated with four, three, and two salt tolerance-related traits, respectively, which explained the high correlation among SSI_VI, SSI_GR_5d, SSI _GR_10d, and SSI_GI (**Figure 1B**).

In this study, 110 and 56 QTNs were co-detected by at least two and three methods, respectively (**Supplementary Table S2** and **Table 2**). Among the 56 QTNs, 12 that were located on chromosomes 1, 2, 3, 6, 8, 9, 11, and 12 were identified to be associated with SSI-GI, of which 11 were identified by ISIS EM-BLASSO, while 10, 9, 8, 7, and 3 were detected by FASTmrMLM, mrMLM, pKWmEB, pLARmEB, and FASTmrEMMA, respectively. Four of the 12 QTNs were simultaneously detected by five methods. Of these four QTNs, rs3_29294598, rs6_30827714, and rs8_24915626, were simultaneously detected by mrMLM, FASTmrMLM, pLARmEB, pKWmEB, and ISIS EM-BLASSO, with PVE values of 2.45 ∼ 5.01, 1.19 ∼ 2.82, and 1.44 ∼ 4.48 (%), respectively. Meanwhile, rs8_27233581 was simultaneously detected by mrMLM, FASTmrMLM, FASTmrEMMA, pKWmEB, and ISIS EM-BLASSO, with a PVE value of 2.28 ∼ 6.28 (%). Six QTNs related to SSI-VI were detected on chromosomes 5, 6, 8, 10, and 11, five of which were identified by mrMLM and pKWmEB, with LOD values of 3.22 ∼ 7.16 and 3.11 ∼ 7.11, respectively. Only one QTN was detected by ISIS EM-BLASSO, with an LOD value of 8.59. Seven QTNs located on chromosomes 1, 2, 4, 6, 9, and 11 were correlated with SSI-MGT. All seven of these QTNs were detected by ISIS EM-BLASSO and pKWmEB, with LOD values of 3.18 ∼ 7.97 and 3.54 ∼ 6.62, respectively. The mrMLM, FASTmrMLM, FASTmrEMMA, and pLARmEB methods detected 3, 5, 1, and 2 QTNs related to SSI-MGT, respectively. Among the seven QTNs, rs1_15357371 was identified by all methods, except for mrMLM, with a PVE value of 2.95 ∼ 5.64 (%). For SSI-IR-24h, four significant QTNs were detected on chromosomes 4, 6, and 9 by mrMLM, pKWmEB, and ISIS EM-BLASSO, with LOD values of 6.97 ∼ 18.97, 3.42 ∼ 7.16, and 3.90 ∼ 10.18, respectively. Two of these QTNs were identified by FASTmrMLM, while none of the QTNs were detected by FASTmrEMMA and pLARmEB. Thirteen QTNs located on chromosomes 1, 2, 3, 4, 6, 7, 10, 11, and 12 were associated with SSI-IR-48h, including 10 that were detected by ISIS EM-BLASSO, with LOD values of 3.54 ∼ 10.0, and nine QTNs that were identified by FASTmrMLM, pLARmEB, and pKWmEB, with LOD values of 3.29 ∼ 6.51, 3.58 ∼ 6.1, and 5.04 ∼ 9.04, respectively. The mrMLM and FASTmrEMMA methods separately detected eight and six QTNs, with LOD values of 3.14 ∼ 6.68 and 3.39 ∼ 6.97, respectively. Of the 13 QTNs, rs1_5453364, rs11_28865880, and rs12_19111880 were identified by all six methods, with PVE values of 0.86 ∼ 2.16, 1.38 ∼ 4.83, and 0.62 ∼ 2.97 (%), respectively. Moreover, 12 QTNs associated with SSI-GR-5d were detected on chromosomes 1, 3, 5, 7, 8, 9, 10, and 11. Of these QTNs, nine, eight, seven, six, six, and four QTNs were separately detected by pLARmEB, FASTmrMLM, mrMLM, pKWmEB, FASTmrEMMA, and ISIS EM-BLASSO, respectively, with LOD values of 3.26 ∼ 7.57, 3.61 ∼ 5.96, 3.03 ∼ 6.43, 3.34 ∼ 6.13, 3.26 ∼ 6.57, and 3.09 ∼ 5.76, respectively. Three of the 12 QTNs, rs3_4264086, rs5_29609065, and rs11_27392033, were detected by five methods, with PVE values of 1.42 ∼ 4.47, 1.07 ∼ 4.65, and 0.96 ∼ 3.86 (%), respectively. For SSI-GR-10d, 12 QTNs were detected on chromosomes 1, 2, 4, 6, 7, 8, 9, 10, and 11. Of these 12 QTNs, rs10_22754603 and rs11_27380577 were identified by five methods, with PVE values of 0.93 ∼ 3.08 and 1.11 ∼ 4.4 (%), respectively (**Table 2**).

**Table 2:** Significant QTNs for SSI-related traits in rice co-detected by at least three multi-locus GWAS methods.

Trait	SNPs^1^	Chromosome	Position	QTN effect	LOD score	PVE (%)^2^	Method^3^
SSI_GI	**rs1_11882948**	1	11882948	0.1 ∼ 0.11	3.67 ∼ 4.68	0.98 ∼ 1.55	2,3,5
	rs2_22250136	2	22250136	-0.16 ∼-0.09	3.27 ∼ 4.43	0.61 ∼ 2.19	1,2,3,4
	rs2_24480757	2	24480757	0.08 ∼ 0.08	3.66 ∼ 4.01	0.98 ∼ 1.5	2,3,6
	**rs3_29294598**	3	29294598	0.08 ∼ 0.17	3.97 ∼ 6.21	2.45 ∼ 5.01	1,2,3,5,6
	rs6_30827714	6	30827714	-0.13 ∼-0.09	3.15 ∼ 6.15	1.19 ∼ 2.82	1,2,3,5,6
	rs8_7832802	8	7832802	0.1 ∼ 0.21	3.48 ∼ 4.94	2.17 ∼ 4.69	1,3,4,6
	**rs8_24915626**	8	24915626	0.09 ∼ 0.16	3.12 ∼ 7.04	1.44 ∼ 4.48	1,2,3,5,6
	rs8_25014297	8	25014297	-0.35 ∼-0.21	5.31 ∼ 10.4	4.56 ∼ 8.91	1,2,3,6
	**rs8_27233581**	8	27233581	0.1∼0.29	3.71 ∼ 7.67	2.28 ∼ 6.28	1,2,3,4,6
	rs9_5893568	9	5893568	0.05 ∼ 0.08	3.17 ∼ 3.43	0.57 ∼ 1.09	2,3,5
	**rs11_17680260**	11	17680260	0.19 ∼ 0.26	6.74 ∼ 10.53	4.9 ∼ 9.8	1,3,5,6
	rs12_21121298	12	21121298	-0.13 ∼-0.09	3.04 ∼ 4.35	1.03 ∼ 2.06	1,2,5
SSI_VI	rs5_29590002	5	29590002	-0.1 ∼ 0.09	3.5 ∼ 4.36	1.26 ∼ 5.77	1,2,4,5
	rs6_26952785	6	26952785	0.1 ∼ 0.11	3.89 ∼ 8.59	3.22 ∼ 3.57	1,3,6
	**rs8_27233581**	8	27233581	0.06 ∼ 0.13	5.12 ∼ 5.45	2.38 ∼ 5.17	1,4,5,6
	rs10_2806159	10	2806159	0.08 ∼ 0.12	3.16 ∼ 5.42	1.85 ∼ 3.55	1,2,6
	**rs10_11718859**	10	11718859	-0.13 ∼ 3.56	4.28 ∼ 6.36	1.74 ∼ 3.2	2,4,5,6
	**rs11_17680260**	11	17680260	0.07 ∼ 0.1	3.12 ∼ 5.68	3.03 ∼7.16	1,5,6
SSI_MGT	rs1_15357371	1	15357371	0.43 ∼ 1.37	4.07 ∼ 6.54	2.95 ∼ 5.64	2,3,4,5,6
	rs2_23991498	2	23991498	0.26 ∼ 0.33	3.06 ∼ 6.62	1.61 ∼ 3.48	2,3,5,6
	rs4_13696726	4	13696726	-0.98 ∼-0.44	3.54 ∼ 6.03	1.79 ∼ 4.88	1,3,6
	rs6_27962052	6	27962052	-0.43 ∼-0.26	3.75 ∼ 4.55	1.06 ∼ 4.81	1,2,3,6
	rs9_4258702	9	4258702	-0.53 ∼-0.41	5.71 ∼ 7.96	2.76 ∼ 3.35	2,3,6
	rs9_11450011	9	11450011	-0.36 ∼-0.31	3.18 ∼ 5.53	1.94 ∼ 3.55	2,3,6
	rs11_24660808	11	24660808	-0.79 ∼-0.44	3.3 ∼ 4.19	2.47 ∼ 2.88	1,3,6
SSI_IR_24h	rs4_31794832	4	31794832	2.59 ∼ 3.07	3.9 ∼ 6.97	0.11 ∼ 2.65	1,3,6
	rs6_5699431	6	5699431	2.33 ∼ 4.08	3.42 ∼ 10.18	0.09 ∼ 6.9	1,2,3,6
	rs9_12353804	9	12353804	-4.57 ∼-3.5	4.95 ∼ 18.97	0.3 ∼ 5.3	1,3,6
	rs9_6746183	9	6746183	-10.84 ∼-4.22	3.29 ∼ 16.12	0.97 ∼ 5.91	1,2,3,6
SSI_IR_48h	rs1_2103242	1	2103242	0.64 ∼ 0.96	3.39 ∼ 6.24	1.46 ∼ 3.92	1,2,4
	rs1_5453364	1	5453364	-1.26 ∼ 0.48	3.23 ∼ 6.79	0.86 ∼ 3.51	1,2,3,4,5,6
	rs1_31748567	1	31748567	-0.83 ∼-0.56	4.04 ∼ 6.43	0.98 ∼ 3.55	1,3,5,6
	rs2_24073194	2	24073194	-0.62 ∼ 0.72	3.58 ∼ 5.04	0.62 ∼ 2.2	3,5,6
	rs3_20204466	3	20204466	0.89 ∼ 1.02	3.81 ∼ 6.73	0.82 ∼ 2.76	3,5,6
	rs4_4695323	4	4695323	-1.5 ∼-0.78	3.29 ∼ 5.61	1.48 ∼ 1.9	2,3,6
	rs4_31202952	4	31202952	-0.67 ∼-0.52	3.43 ∼ 5.63	1.61 ∼ 2.37	1,2,3
	rs6_1459330	6	1459330	0.62 ∼ 1.1	3.34 ∼ 5.38	1.51 ∼ 1.71	1,2,4
	rs7_21649301	7	21649301	1.08 ∼ 1.6	4.4 ∼ 6.08	1 ∼ 3.78	1,2,5
	rs10_10209541	10	10209541	0.67 ∼ 1.03	3.54 ∼ 6.61	1.51 ∼ 3.98	3,5,6
	rs11_28865880	11	28865880	0.7 ∼ 1.65	5.83 ∼ 10	1.38 ∼ 4.83	1,2,3,4,5,6
	rs12_7176832	12	7176832	-1.43 ∼-0.84	4.43 ∼ 8.64	1.2 ∼ 4.91	2,3,4,5,6
	rs12_19111880	12	19111880	-1.66 ∼-0.66	3.14 ∼ 7.23	0.62 ∼ 2.97	1,2,3,4,5,6
SSI_GR_5d	**rs1_11882948**	1	11882948	0.12 ∼ 0.15	3.03 ∼ 3.94	1.69 ∼ 1.99	1,2,6
	rs1_22648607	1	22648607	0.17 ∼ 0.23	4.49 ∼ 6.57	1.36 ∼ 1.85	3,5
	rs3_4264086	3	4264086	0.11 ∼ 0.21	3.97 ∼ 6.43	1.42 ∼ 4.47	1,2,4,5,6
	**rs3_29294598**	3	29294598	0.1 ∼ 0.11	3.59 ∼ 3.61	1.34 ∼ 1.72	2,5
	rs5_29609065	5	29609065	0.08 ∼ 0.22	3.34 ∼ 5.74	0.96 ∼ 3.86	1,2,4,5,6
	rs7_1171356	7	1171356	0.15 ∼ 0.28	4.48 ∼ 4.77	3.26 ∼ 3.58	1,4
	**rs8_24915626**	8	24915626	0.11 ∼ 0.15	3.31 ∼ 4.1	1.5 ∼ 3.01	2,3,5
	**rs8_27233581**	8	27233581	0.09 ∼ 0.28	3.09 ∼ 4.66	1.36 ∼ 3.33	3,4,6
	rs9_8174432	9	8174432	0.1 ∼ 0.15	3.26 ∼ 4.23	1.29 ∼ 2.84	1,2,5
	**rs9_21139613**	9	21139613	0.11 ∼ 0.19	3.58 ∼ 5.02	1.61 ∼ 4.79	1,5,6
	**rs10_11718859**	10	11718859	-0.23 ∼-0.11	4.32 ∼ 5.76	1.17 ∼ 2.5	2,3,4,5
	rs11_27392033	11	27392033	-0.31 ∼ 0.12	3.93 ∼ 6.37	1.07 ∼ 4.65	1,2,4,5,6
SSI_GR_10d	rs1_3401561	1	3401561	-0.24 ∼-0.14	3.21 ∼ 4.65	2.29 ∼ 6.08	1,3,5
	**rs1_11882948**	1	11882948	0.1 ∼ 0.12	3.35 ∼ 4.56	1.31 ∼ 1.72	2,3,6
	rs2_8009453	2	8009453	0.08 ∼ 0.15	3.34 ∼ 4.96	1.18 ∼ 3.31	1,2,3
	rs2_22247315	2	22247315	-0.17 ∼-0.12	3.54 ∼ 6.51	0.95 ∼ 3.19	3,5,6
	rs4_19568498	4	19568498	0.16 ∼ 0.21	5.39 ∼ 6.37	1.9 ∼ 2.96	2,3,6
	rs6_26597879	6	26597879	0.11 ∼ 0.13	3.83 ∼ 5.41	2.44 ∼ 2.82	2,3,5
	rs7_3788168	7	3788168	0.22 ∼ 0.23	5.46 ∼ 7.54	2.54 ∼ 3.59	2,3,6
	rs7_22276671	7	22276671	0.09 ∼ 0.1	3.65 ∼ 4.66	1.57 ∼ 2.33	2,3,6
	**rs8_24915626**	8	24915626	0.13 ∼ 0.14	3.88 ∼ 5.8	2.07 ∼ 3.16	1,2,3
	**rs9_21139613**	9	21139613	0.1 ∼ 0.18	3.55 ∼ 8.69	1.92 ∼ 4.59	1,2,5,6
	rs10_22754603	10	22754603	0.08 ∼ 0.17	3.46 ∼ 5.94	0.93 ∼ 3.08	1,2,3,5,6
	rs11_27380577	11	27380577	-0.21 ∼-0.13	3.24 ∼ 7.31	1.11 ∼ 4.4	1,2,3,5,6


*^1^SNPs in bold font are pleiotropic QTNs which were detected associate with multiple traits.*

*^2^PVE: Phenotypic variation explained.*

*^3^1:mrMLM; 2:FASTmrMLM; 3:ISIS EM-BLASSO; 4:FASTmrEMMA; 5:pLARmEB; 6:pKWmEB.*

### Validation of the Common QTNs

**FIGURE 2:**
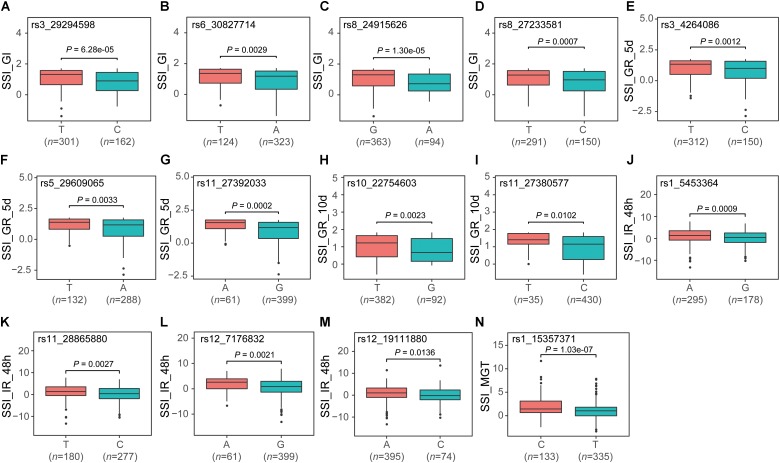
Boxplot for validating 14 co-detected QTNs **(A–N)**. For each QTN, the population was divided into two groups according to allele types. The X-axis represents the two alleles for each QTN, while the Y-axis corresponds to the phenotype.

Among the 56 QTNs, 14 were identified by at least five methods, of which four, three, two, four, and one were associated with SSI_GI, SSI_GR_5d, SSI_GR_10d, SSI_IR_48h, and SSI_MGT, respectively. We divided the population into two groups according to allelic genotypes to test whether the mean phenotypes of the two groups were significantly different. The mean value of the group carrying the favorable allele was less than that of the other group (**Figure 2**).

### GO and KEGG Pathway Enrichment Analyses

According to the Nipponbare reference genome, the 371 identified QTNs for traits related to salt tolerance were part of or were adjacent to 581 genes (**Supplementary Table S1**). These genes were significantly enriched for GO biological processes related to the plant lipid metabolic process and transmembrane transport process (**Supplementary Table S3**). They were also significantly enriched for the plant tryptophan metabolism pathway (*P* < 0.03). Moreover, two genes (LOC_Os01g45760 and LOC_Os10g04860) were associated with auxin biosynthesis. A total of 66 genes were identified around the 56 QTNs based on the enriched GO terms and KEGG pathways as well as the functional annotations (**Supplementary Table S4**). This information may be very useful for identifying the genes responsible for salt tolerance in rice.

## Discussion

Multi-locus GWAS models, which are relatively close to the true genetic models of plants and animals, are superior to single-locus GWAS models because of their higher statistical power and lower FPR (Segura et al., 2012; Wang et al., 2016a). These models were developed by geneticists, who added the polygenic effect and population structure to the single-locus GWAS model to decrease the bias in effect estimations by controlling the genetic background (Zhang et al., 2005; Yu et al., 2006; Zhang et al., 2010). Although advancements in the single-locus GWAS models have improved the detection accuracy to some extent, the multiple test correction for the threshold value of the significance test in single-locus models (e.g., Bonferroni correction) is too stringent to capture all true QTNs. Another unavoidable problem is that single-locus GWAS methods are inappropriate when the target traits are controlled by a series of polygenes. In this study, 478 rice accessions with 162,529 SNPs were used to identify QTNs for traits related to salt tolerance based on six multi-locus GWAS methods. We compared the QTNs identified by the multi-locus GWAS methods in our study with the previously reported QTNs detected by the efficient mixed-model EMMA eXpedited (EMMAX) program comprising a single-locus GWAS method. The comparison revealed that four of the previously reported six QTNs related to SSI-VI were detected by a multi-locus GWAS, and two QTNs associated with SSI-MGT overlapped with the previously reported QTNs. Additionally, 12, 4, 13, 12, and 12 QTNs separately associated with SSI-GI, SSI-IR-24h, SSI-IR-48h, SSI-GR-5d, and SSI-GR-10d, respectively, were simultaneously detected by at least three multi-locus GWAS methods. In contrast, none of the QTNs associated with the five traits were identified by a single-locus GWAS method. These observations were as expected, and can be explained by the following two points: (i) salt tolerance is a quantitative genetic characteristic that is controlled by multiple genes with small effects, which are difficult to detect in a single-locus GWAS model (Wang et al., 2011; Kumar et al., 2015); (ii) some true QTNs for traits related to salt tolerance are missed by a single-locus GWAS model because of an overly conservative critical value. Furthermore, our results suggest that a multi-locus GWAS model may be useful for detecting loci with small effects.

In this study, we used six multi-locus GWAS methods included in the mrMLM package to detect QTNs. The six methods involve two-step algorithms, and marker effects are treated as random effects in each method. However, each method has its own characteristics. We observed that mrMLM detected the most QTNs (**Supplementary Table S1**), but this method has one shortcoming. When the number of putative QTNs is much larger than the sample size, the multi-locus model in this method will be over-fitted. The residual error estimated by mrMLM was much smaller than that estimated by the five other methods (**Table 1**). During the first step, 7,588 QTNs with a threshold value *P* < 0.01 were selected, which is 16 times larger than the sample size. Over-fitting may occur when too many variables are added to a multi-locus model. This issue was solved by using FASTmrMLM, in which the least angle regression (LARS) algorithm is implemented between the first single-locus scanning step and the EM-Empirical Bayes estimation in the second step. The LARS algorithm (Efron et al., 2004) is a flexible method for selecting variables, and can be applied in the lars package^1^. In this method, *n*-1 variables (*n* is the number of samples), which are most likely associated with the target traits, are added to the multi-locus model.

The FASTmrEMMA method detected the fewest QTNs. This method involves an approximation algorithm in which the covariance matrix of the polygenic matrix *K* and environmental noise are whitened by a matrix transformation to increase the computing speed. In the pLARmEB method, the same transformed model as that used in FASTmrEMMA is implemented to control the polygenic background, and the LARS algorithm is applied to select potential SNPs related to the target trait for the subsequent multi-locus GWAS detection. Among the six multi-locus GWAS methods, ISIS-EM-BLASSO had the shortest running time and the smallest estimated residual errors (**Supplementary Figure S2** and **Table 1**). In the first step of this method, an iterative-modified sure independence screening (ISIS) approach is used to decrease the number of SNPs to a moderate level, after which the Expectation-Maximization (EM)-Bayesian least absolute shrinkage and selection operator (BLASSO) is used to estimate all of the selected SNP effects to detect true QTNs. The last method, pKWmEB, is a non-parametric method, in which a Kruskal–Wallis test and the LARS algorithm are used to identify potential SNPs. All identified markers are added to the multi-locus model to detect true QTNs.

The two-step multi-locus GWAS methods included in this study significantly improved the statistical power and decreased the FPR. Moreover, ISIS EM-BLASSO identified the most co-detected QTNs, followed by pKWmEB, while FASTmrEMMA identified the fewest QTNs (**Table 2**). Additionally, ISIS EM-BLASSO performed best, with the smallest estimated residual errors and highest computing speed. However, selecting an appropriate critical value is still problematic for the two-step multi-locus GWAS model. A threshold value that is too stringent will lead to the omission of loci information, whereas a relaxed threshold value will result in numerous loci being selected, which may lead to the over-fitting of multi-locus models. A simple solution to this problem involves developing a hybrid method that combines the results from different methods. Directly decreasing the number of SNPs instead of applying a single-locus GWAS scanning step represents another potential solution. We are currently developing a new bin analysis method that can be applied to any type of population. In the bin analysis method, the number of markers is decreased, but the information for all markers is fully retained. Adding a bin analysis to the multi-locus GWAS model represents a new option.

## Conclusion

In this study, six multi-locus GWAS methods were used to detect loci related to rice salt tolerance at the seed germination stage. A total of 371 QTNs were identified, with 56 QTNs co-detected by at least three methods. Moreover, 66 genes were identified in the vicinity of the 56 QTNs based on functional annotations. Two of these genes (LOC_Os01g45760 and LOC_Os10g04860) are involved in auxin biosynthesis according to the enriched GO terms and KEGG pathways. These observations may be useful for identifying the genes responsible for rice salt tolerance.

## Author Contributions

YC drafted the manuscript. FZ and YC analyzed the data. YZ and FZ conceived the study and were in charge of the direction and planning. All authors read and approved the final version of this manuscript.

## Conflict of Interest Statement

The authors declare that the research was conducted in the absence of any commercial or financial relationships that could be construed as a potential conflict of interest.

## Footnotes

1 http://cran.r-project.org/web/packages/lars/

## Supplementary Material

FIGURE S1 Bar plot of the number of QTNs associated with seven salt tolerance traits detected by different methods.

FIGURE S2 Computing time of the six multi-locus GWAS methods.

TABLE S1 Significant salt tolerance QTNs detected by six multi-locus GWAS methods.

TABLE S2 Significant QTNs detected by at least two multi-locus GWAS methods.

TABLE S3 Results of a GO enrichment analysis.

TABLE S4 Gene annotations for the 56 significant QTNs associated with salt tolerance traits.

## Abbreviations

GI: germination index
GR-10d: germination rate at the 10th day
GR-5d: germination rate at 5th day
IR-24h: imbibition rate at 24 h
IR-48h: imbibition rate at 48 h
MGT: mean germination time
N: normal condition
S: salt stress condition
SSI: stress-susceptibility index
VI: vigor index
