# Supplementary figures and images for: The Application of Multi-Locus GWAS for the Detection of Salt-Tolerance Loci in Rice
Source: Front Plant Sci. 2018 Oct 4;9:1464. doi: 10.3389/fpls.2018.01464 (PMC6180169; doi:10.3389/fpls.2018.01464)

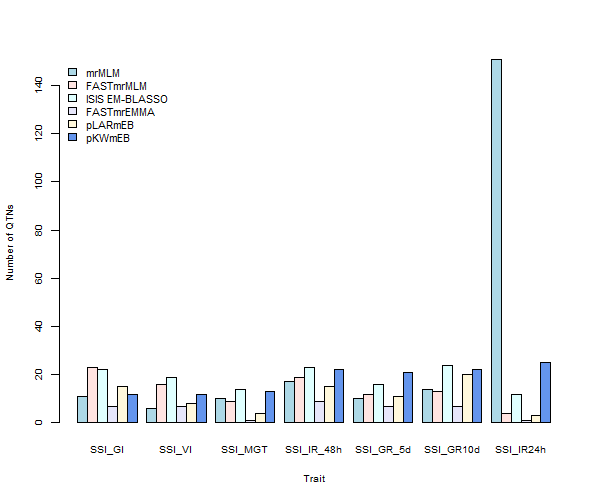

Supplement: FIGURE S1 — Bar plot of the number of QTNs associated with seven salt tolerance traits detected by different methods. [file Image_1.TIFF]

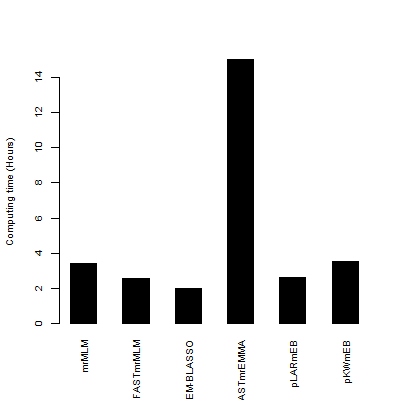

Supplement: FIGURE S2 — Computing time of the six multi-locus GWAS methods. [file Image_2.TIFF]
